# Limited Role of Hair Cortisol and Cortisone Measurement for Detecting Cortisol Autonomy in Patients With Adrenal Incidentalomas

**DOI:** 10.3389/fendo.2022.833514

**Published:** 2022-02-08

**Authors:** Soraya Puglisi, Marta Leporati, Eleonora Amante, Alice Parisi, Anna Rosa Pia, Paola Berchialla, Massimo Terzolo, Marco Vincenti, Giuseppe Reimondo

**Affiliations:** ^1^ Internal Medicine, Department of Clinical and Biological Sciences, San Luigi Gonzaga Hospital, University of Turin, Turin, Italy; ^2^ Centro Regionale Antidoping e di Tossicologia “A. Bertinaria”, Turin, Italy; ^3^ Department of Chemistry, University of Turin, Turin, Italy; ^4^ Statistical Unit, Department of Clinical and Biological Sciences, University of Turin, Turin, Italy

**Keywords:** subclinical Cushing, hair glucocorticoids, screening, diagnosis, adrenal adenoma, adrenal incidentaloma, Cushing, UPLC-MS/MS

## Abstract

Several studies demonstrated the diagnostic accuracy of hair glucocorticoid measurement in patients with overt Cushing syndrome, but few data are available for patients with adrenal incidentaloma (AI) and cortisol autonomy. The aim of our study was to assess whether measurement of 5 corticosteroid hormones with the ultra-high-performance liquid chromatography-tandem mass spectrometry (UPLC-MS/MS) method in the keratin matrix is useful to stratify patients with AI by the presence of autonomous cortisol secretion [ACS] (defined as serum cortisol after 1 mg dexamethasone suppression test (DST) > 138 nmol/l) or possible ACS [PACS] (defined as serum cortisol after 1 mg DST > 50 nmol/l but ≤138 nmol/l). We analysed data of 67 AI patients (32 with cortisol autonomy) and 81 healthy subjects. We did not find any significant statistical difference comparing hair cortisol, cortisone, and 20β-dihydrocortisol concentrations between healthy controls and AI patients, while 6β-hydroxycortisol and 11-deoxycortisol were undetectable. Moreover, no significant difference was found in hair cortisol, cortisone, and 20β-dihydrocortisol levels of AI patients with or without cortisol autonomy. Finally, we did not find any correlation in patients with AI between hormonal concentrations in the keratin matrix and serum, salivary, and urinary cortisol levels, or with body mass index. In conclusion, our findings suggest that hair glucocorticoid measurement is not suitable as a diagnostic test for cortisol autonomy (ACS and PACS).

## Introduction

Adrenal incidentalomas (AIs) are masses incidentally and unexpectedly found in patients who undergo radiological exams due to diagnostic process or follow-up of extra-adrenal diseases (1). Since in the last decades the use of high-resolution cross-sectional imaging has become increasingly widespread, the serendipitous detection of adrenal tumours is common in clinical practice, accounting for 4.2%–7.3% in recent computed tomography (CT) series, up to 10% in elder patients (2–5).

In most cases, AI are benign adenomas, which secrete cortisol autonomously in up to 50% of patients (6, 7). In many cases, cortisol excess is mild and patients present without a typical phenotype, a condition previously called subclinical Cushing (8, 9) and now defined as autonomous cortisol secretion (ACS) or possible ACS (PACS) (1). Recent studies have demonstrated that this chronic, low-grade hypercortisolism can be associated with several cardio-metabolic comorbidities (5, 10–15) and increased mortality (16–18).

However, the diagnosis of ACS and PACS is challenging in practice and it can remain unrecognized for a long time, due to the subtle and heterogeneous clinical presentation and some methodological issues in laboratory screening tests (6).

According to the European Society of Endocrinology (ESE) and the European Network for the Study of Adrenal Tumors (ENSAT) guidelines, the main diagnostic tool is the 1-mg overnight dexamethasone suppression test (DST): cortisol levels ≤ 50 nmol/l exclude ACS, cortisol levels > 138 nmol/l define ACS, and levels between these two thresholds are considered expression of PACS (1). However, false positive or false negative results can occur, due to interferences in the absorption and/or the metabolism of dexamethasone (i.e., drugs, liver, or renal failure). The 24-h urinary free cortisol (UFC), midnight salivary cortisol (MSC), and plasma ACTH measurement at 8.00 a.m. support the diagnosis, but these tests are prone to several analytical errors or interference (6). Moreover, all these tools present the remarkable limit of measuring cortisol concentration at a single time point. Therefore, they cannot give an adequate representation of a chronic exposure to cortisol excess.

To overcome this issue, the measurement of hair cortisol has been recently proposed, given that cortisol progressively accumulates in hair shaft by passive diffusion from blood capillaries, according to the hair grow rate of approximately 1 cm per month. Cortisol remains sequestered in the keratin matrix with relatively little degradation over time, providing a window of detection which is much wider (weeks to several months) than that of serum or urine, in which cortisol levels decrease rapidly over a relatively short period of time (hours to days).

Although several studies confirmed the diagnostic accuracy of hair cortisol in patients with overt Cushing syndrome (19–23), only one study analysed the measurement of hair cortisol and cortisone in patients with AI (24).

In our study, we employed the method of the ultra-high-performance liquid chromatography-tandem mass spectrometry (UPLC-MS/MS) for the simultaneous measurement of 5 corticosteroid hormones in the keratin matrix in AI patients with or without cortisol autonomy (ACS and PACS) and in healthy controls.

## Materials and Methods

### Participants

We assessed consecutive patients affected by AI, which referred to the Unit of Internal Medicine at San Luigi Gonzaga Hospital, Orbassano (Italy), between March 2019 and February 2020. We compared this cohort with a group of healthy subjects, matched by sex and age with the AI group. Data of AI patients and healthy control were obtained from their medical records, interviews, and physical examinations and were reported on a detailed computerized database. Both patients and controls voluntarily participated in this study and gave written informed consent to the collection of data according to the local ethics committee indications (Registry and Repository of biological samples of ENSAT).

The inclusion criteria for AI patients were diagnosis of cortical adrenal adenoma, with specific CT characteristics (size less than 4 cm with well-defined margins, homogeneous and hypodense content). The exclusion criteria were suspected pheochromocytoma (i.e., patients with high levels of free plasma metanephrines or high urinary fractionated metanephrines), suspected primary hyperaldosteronism (i.e., patients with severe or resistant hypertension, hypokalemia, increased aldosterone/renin ratio), or suspected overt Cushing (i.e., patients with clinical signs of hypercortisolism or hyperandrogenism); patients who used topical or systemic corticosteroids in the last 3 months; and history of malignancy (breast, lung, and kidney cancer or melanoma) which can frequently metastasize in the adrenal gland.

The exclusion criteria for the healthy subjects were history of adrenal masses, Cushing syndrome, history of malignancy, suspected pseudo-Cushing states (i.e., chronic alcoholism, psychiatric disorders, poorly controlled diabetes mellitus, etc.), shift workers, and topical or systemic use of corticosteroids in the last 3 months.

Medical charts were reviewed to obtain clinical information: age, sex, body mass index (BMI), waist, and adrenal tumour size. Overweight was defined by BMI ≥ 25 and ≤ 30 kg/m^2^, and obesity was defined by BMI > 30 kg/m^2^. In all AI patients, we reported cortisol after 1 mg DST, 24-h UFC, and plasma ACTH at 8.00 a.m. and, when available, MSC.

### Hair Cortisol Assessment

The determination and quantification of the analytes (cortisol, its metabolites cortisone, 20β-dihydrocortisol, and 6β-hydroxycortisol, and its precursor 11-deoxycortisol) in hair were carried out by the Regional Anti-Doping Center “Alessandro Bertinaria” (CAD), Orbassano (Turin). Methods of sample collection and UPLC-MS/MS analysis were similar to those commonly employed in toxicology and were adapted to the evaluation of the five targeted steroids (25).

### Chemicals, Reagents, and Standard Solutions

Cortisol, cortisone, 20β-dihydrocortisol, 6β-hydroxycortisol and 11β-deoxycortisol, acetonitrile (ACN), methanol (MeOH), and dichloromethane were provided by Sigma-Aldrich (Milan, Italy). Cortisol-d2 was purchased by C/D/N Isotopes Inc. (Pointe-Claire, Quebec, Canada). Acetic acid was purchased by Carlo Erba (Cornaredo, MI, Italy). Ultrapure water was obtained by a Milli-Q Millipore system (Bedford, MA, USA). Stock standard solutions of analytes and internal standard (IS), cortisol d2, were prepared in MeOH at a concentration of 1 mg/ml and stored at −20°C in the dark. Working MeOH solutions containing all the analytes at different concentrations were prepared by mixing the stock solutions at the proper dilution. The working solutions were used to spike negative hair samples at various levels.

### Hair Sampling

Two small strands of hair were collected from the posterior vertex as close as possible to the scalp, according to the *Society of Hair Testing* and literature recommendations (26). For the analysis, we used the most proximal 3 cm of hair, corresponding to the most recent 3 months.

### Analytical Method

**Figure 1:**
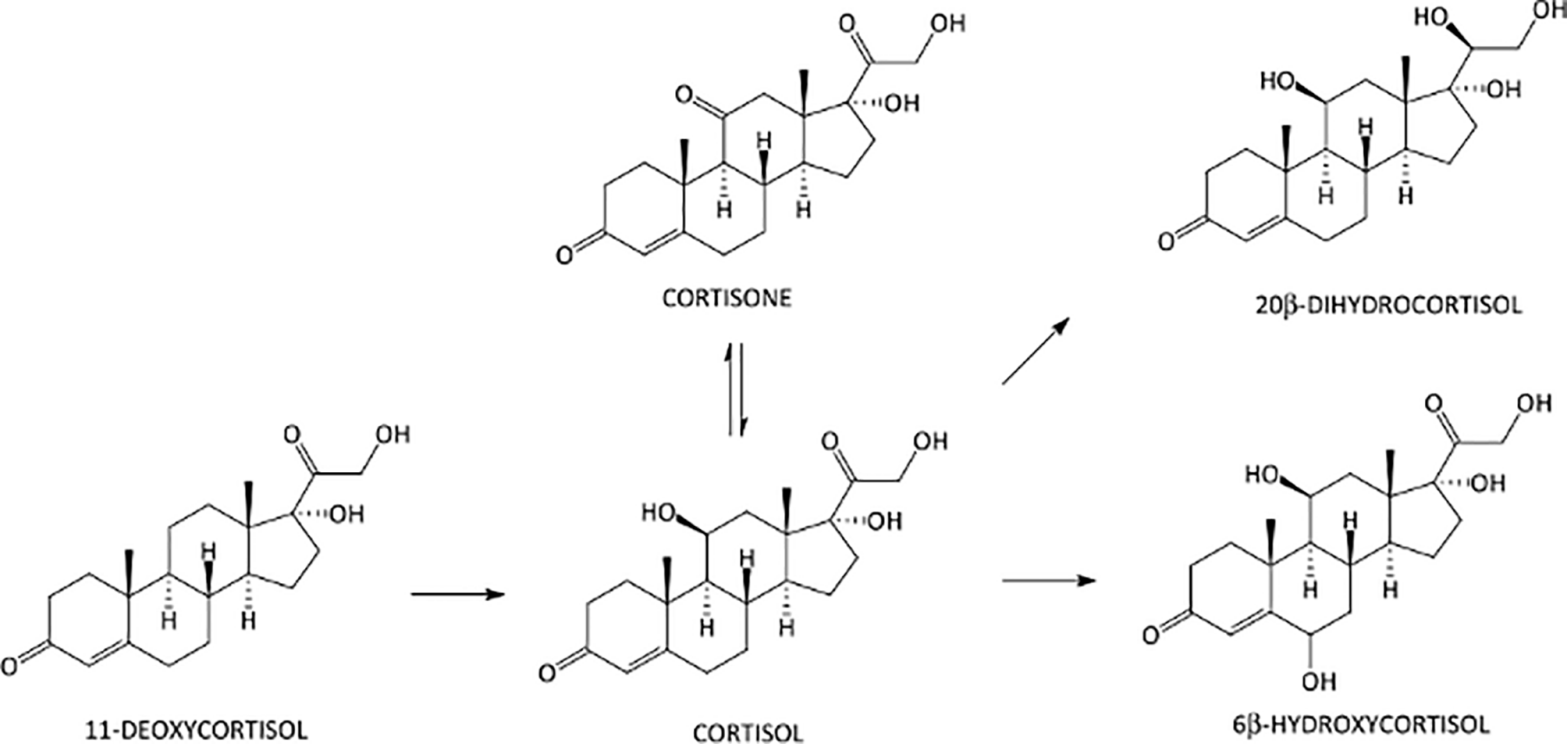
Chemical structures of the steroids that were measured in the hair.

All analyses were performed on a Shimadzu Nexera 30 UPLC-system (Shimadzu, Duisburg, Germany) interfaced to an AB Sciex API 5500 triple quadrupole mass spectrometer (AB Sciex, Darmstadt, Germany) with an electrospray Turbo Ion source operating in the negative (ESI–) ion mode. An Acquity UPLC BEH C18 column 100 mm × 2.1 mm, 1.7 μm (Waters Corporation, Milan, Italy), protected by a C18 VanGuard pre-column, was used for the separation of the target analytes. The column oven was maintained at +40°C, and the elution solvents used were aqueous 0.1% aqueous acetic acid (solvent A) and ACN acidified with 0.1% of acetic acid (solvent B). The mobile phase was eluted under the following conditions (a/b; v/v): initial 90:10 ratio for 1 min, linear gradient to 55:45 in 6 min, linear gradient to 10:90 in 0.5 min, and final isocratic condition at 90% B for 0.5 min. The flow rate was 0.4 ml/min, and total run time was 8 min, plus 4 min of re-equilibration time. The MS system was operated in the selected reaction monitoring mode (SRM). In order to establish appropriate SRM conditions, each analyte was individually infused into the ESI capillary, while the declustering potential (DP) and the entrance potential (EP) were adjusted to maximize the intensity of the parent ions, the adducts of the corticosteroid molecules with acetic acid [M+CH3COO]^–^ species. The collision offset voltage (CE) was adjusted to preserve approximately 10% of the precursor ion, and the cell exit potentials (CXP) were also optimized. The best results were obtained using a source block temperature of +500°C and an ion-spray voltage of -4.500 V. Both Q1 and Q3 were operated at unit mass resolution. Nitrogen was employed as the collision gas. The gas settings were as follows: curtain gas 35.0 psi, collision gas 8.0 psi, ion source gas GS1 40.0 psi, and ion source gas GS2 40.0 psi. Analyst 1.5.2 (AB Sciex) software was used for data processing. The chemical structures and biochemical interactions of the glucocorticoids investigated in this study are reported in **Figure 1**. All analytes and IS and their corresponding retention time, SRM transitions, and potentials are presented in **Table 1**.

**Table 1:** Mass spectrometry settings for the target compounds and the internal standard Cortisol-d2.

Analyte	t_r_ (min)	Precursor ion	DP (V)	EP (V)	Target fragment	CE (V)	CXP (V)	Qualifier fragment	CE (V)	CXP (V)
6β-Hydroxycortisol	2.96	437.2	-45	-12	347.3	-23	-13	313.4	-45	-10
20β-Dihydrocortisol	5.32	423.2	-56	-13	333.3	-35	-12	363.5	-35	-12
Cortisol	5.79	421.1	-57	-3	330.9	-24	-10	296.6	-43	-10
Cortisone	5.83	419.2	-58	-7	328.9	-25	-10	300.8	-30	-12
11-Deoxycortisol	6.84	405.3	-46	-12	315.3	-24	-13	345.4	-12	-13
Cortisol-d2	5.76	423.1	-69	-13	332.9	-38	-13	–	–	–

The values of the declustering potential (DP), exit potential (EP), collision offset voltage (CE), and cell exit potential (CXP) are reported.

### Sample Preparation

About 200 mg of hair was twice-washed, once with dichloromethane and once with methanol (1.5 ml, vortex mixed for 3 min). After complete removal of solvent wash, the hair was dried at room temperature by a gentle nitrogen flow and subsequently cut with scissors into 1–2-mm segments. An aliquot of about 50 mg was weighted and then fortified with 25 µl of an IS working solution at 10 ng/ml, yielding a final concentration of 5 pg/mg. Sample extraction was carried out by addition of 1 ml of methanol, vortex shaking for 5 min, and centrifuging at 4,000 rpm for 3 min, to ensure the complete immersion of the matrix into the solvent, and final incubation at 55°C for 15 h. Lastly, 100 µl of the organic phase was collected and evaporated to dryness under a gentle stream of nitrogen and mild heating (25°C) using a Techne Sample Concentrator (Barloworld Scientific, Stone, UK). The residue was dissolved in 100 µl of mixture of the initial mobile phase of the following chromatographic run, transferred into a vial, and centrifuged at 4,000 rpm for 10 min. 10 µl of solution was injected into the UPLC–MS/MS system.

### Validation

The analytical method was validated in accordance with the criteria and recommendations of international guidelines (27). The following parameters were investigated: specificity, selectivity, calibration range and model, detection and quantification limits (LOD and LOQ), and intra-assay and inter-assay precision and accuracy. Carryover and matrix effect were also investigated. Details about the validation procedure can be found elsewhere (28, 29). All the validation parameters resulted within the ranges of acceptability. In particular, lack-of-fit test and Mandel’s test confirmed (95% confidence) the linearity of the calibration models for all steroids over the calibration range. Accuracy was verified at low, intermediate, and high concentrations, with bias% values largely below 15%. Intra-assay and inter-assay precision tests yielded coefficients of variation (CV%) below 10%. Calibration range, LOD and LOQ values, inter-day trueness, and inter-day precision are reported in **Table 2**.

**Table 2:** Method’s validation parameters, including calibration range, adjusted R^2^, limit of detection (LOD), limit of quantitation (LOQ), inter-day trueness (bias %), and inter-day precision (CV%).

Analyte	Calibration range (ng/g)	Determination coefficient (adj R^2^)	LOD (ng/g)	LOQ (ng/g)	Bias % 0.5 ng/g *^a^*	Bias % 5 ng/g *^a^*	Bias % 25 ng/g *^a^*	CV % 0.5 ng/g	CV % 5 ng/g	CV % 25 ng/g
6β-Hydroxycortisol	0.5–50	0.9990	0.2	0.5	93.3%	100.8%	95.3%	9.1%	6.4%	6.0%
20β-Dihydrocortisol	0.5–50	0.9963	0.2	0.7	103.0%	102.2%	97.0%	7.0%	8.6%	5.1%
Cortisol	0.5–50	1.0000	0.1	0.4	100.2%	104.6%	97.6%	9.0%	6.5%	5.2%
Cortisone	0.5–50	0.9996	0.1	0.3	98.4%	103.9%	98,3%	9.6%	6.8%	6.4%
					5 ng/g	25 ng/g	50 ng/g	5 ng/g	25 ng/g	50 ng/g
11-Deoxycortisol	2–50	0.9931	0.9	2.7	102.9%	104.5%	97.0%	7.2%	7.4%	5.6%

Inter-day trueness and precision were calculated from n. 30 repeated analyses for each concentration level executed during a period of 27 days.

a Coincidence with the expected value corresponds to 100%.

### Statistical Analysis

The occurrence of bias due to age or sex was initially investigated in the samples from healthy individuals. The Spearman correlation coefficient was computed on the levels of corticosteroids from individuals of the same sex along the sampled age range (20–83 years). The sex bias was investigated using the analysis of variance (ANOVA), and a significance threshold of 0.05 was chosen. The analysis demonstrated the absence of bias for these parameters.

The concentrations of the compounds in healthy individuals and patients with adrenal incidentaloma were compared using the Mann–Whitney or Kruskal–Wallis test as appropriate.

The group sizes (81 healthy subjects and 67 AI patients) achieved about 83% power to detect a difference in hair cortisol of at least 2.5 pg/mg (2.5 in healthy patients vs. 5 in AI patients) using a two-sided Mann–Whitney U or Wilcoxon rank-sum test when the significance level (alpha) of the test was 0.050 and the standard deviation was 5.0 in both groups.

The statistical calculations used in the analytical method validation were made by an *ad-hoc* Excel^®^ sheet built in-house to adapt a published R-routine. The details are reported elsewhere (29).

## Results

We collected data from 81 healthy subjects and 67 patients with AI. The baseline characteristics are reported in **Table 3**.

**Table 3:** Baseline characteristics of healthy subjects and patients with AI.

Characteristics	Healthy controls N° 81	AI patients N° 67	*p* value
**Gender**			0.10
Male, *N* (%)	48 *(59.3)*	30 *(44.8)*	
Female, *N* (%)	33 *(40.7)*	37 *(55.2)*	
**Age**, year			0.22
Median (IQR)	64 (58–71)	68 (61–74)	
**BMI**, kg/m^2^			/
Median (IQR)	/	27.1 (25.6–31.0)	
**Waist**, cm			/
Median (IQR)	/	102 (90.5–109.5)	
**Cortisol after 1 mg-DST,** nmol/L			/
Median (IQR)	/	49 (35–64)	
**24-h UFC,** nmol/24 h			/
Median (IQR)	/	185 (148–257)	
ULN median (IQR)	/	0.8 (0.7–1.2)	
**MSC,** nmol/L			/
Median (IQR)	/	11 (7–13)	
ULN median (IQR)	/	1.3 (0.8–1.6)	
**ACTH,** pmol/L			/
Median (IQR)	/	3.5 (2.4–5.4)	
**Adrenal tumor size,** mm			/
Median (IQR)	/	24 (18.5–30)	
**Hair cortisol levels,** pg/mg			0.26
Median (IQR)	3.40 (1.80–6.88)	2.72 (1.24–6.78)	
**Hair cortisone levels,** pg/mg			0.25
Median (IQR)	13.21 (9.94–22.00)	10.53 (6.43–22.91)	
**Hair 20β-dihydrocortisol levels,** pg/mg			0.39
Median (IQR)	1.55 (0.51–2.63)	1.63 (0.98–3.36)	
**Ratio hair cortisol/cortisone levels**			0.18
Median (IQR)	0.26 (0.18–0.36)	0.22 (0.14–0.34)	

AI, adrenal incidentalomas; N, number of patients; IQR, interquartile range; BMI, body mass index; DST, dexamethasone suppression test; UFC, urinary free cortisol; MSC, midnight salivary cortisol; ACTH, adrenocorticotropic hormone.

**Figure 2:**
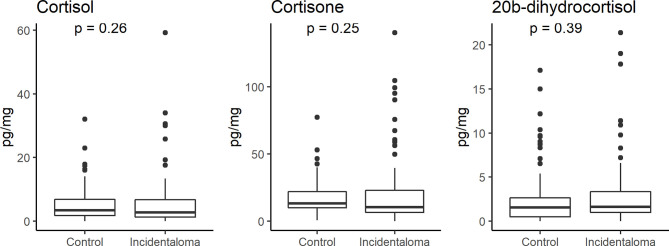
Comparison of hair cortisol, cortisone, and 20β-dihydrocortisol levels between healthy controls and patients with adrenal incidentaloma showing the lack of any significant difference.

No significant statistical difference was found in the hair concentrations of cortisol, 20β-dihydrocortisol, and cortisone between controls and patients (median 3.40, IQR 1.80–6.88, vs. 2.72, 1.24–6.78, pg/mg; median 1.55, 0.51–2.63, vs. 1.63, 0.98–3.36, pg/mg; median 13.21, 9.94–22.00, vs. 10.53, 6.43–22.91, pg/mg, respectively) (**Figure 2**). 6β-Hydroxycortisol and 11-deoxycortisol were undetectable in all samples.

Among the AI patients, 35 had cortisol values ≤ 50 nmol/l (excluding cortisol autonomy), while the remainders had biochemical evidence of cortisol autonomy, either ACS (n = 3) or PACS (n = 29). The baseline characteristics of the two groups are reported in **Table 4**.

**Table 4:** Comparison of baseline characteristics of AI patients, according to the presence of cortisol autonomy.

Characteristics	AI patients without cortisol autonomy N° 35	AI patients with cortisol autonomy N° 32	*p* value
**Sex**			0.74
Male, *N* (%)	15 *(42.9)*	15 *(46.9)*	
Female, *N* (%)	20 *(57.1)*	17 *(53.1)*	
**Age**, year			**<0.001**
Median (IQR)	64 (58–70)	70.5 (68–75)	
**BMI**, kg/m^2^			
Median (IQR)	27.1 (25.9–30.3)	27.5 (25.4–31.4)	0.81
*Normal*	5 *(14.3)*	7 *(21.9)*	
*Overweight*	20 *(57.1)*	12 *(37.5)*	
*Obese*	10 *(28.6)*	13 *(40.6)*	
**Waist**, cm			0.14
Median (IQR)	100 (89.5–105)	104.5 (92–115)	
**Cortisol after 1 mg-DST,** nmol/L			**<0.01**
Median (IQR)	35 (28–41)	69 (59–91)	
**24-h UFC,** nmol/24 h			0.66
Median (IQR)	188 (145–248)	185 (158–268)	
ULN median (IQR)	0.9 (0.7–1.1)	0.8 (0.7–1.2)	
**MSC,** nmol/L			0.62
Median (IQR)	10 (7–14)	11 (7–13)	
ULN median (IQR)	1.3 (0.8–1.6)	1.3 (0.9–1.6)	
**ACTH,** pmol/L			**0.03**
Median (IQR)	3.9 (3.2–5.7)	3.0 (1.8–4.3)	
**Adrenal tumor size,** mm			**<0.01**
Median (IQR)	20 (16–24)	30 (24–36)	
**Hair cortisol levels,** pg/mg			0.51
Median (IQR)	2.33 (1.16–6.54)	2.97 (1.38–7.40)	
**Hair cortisone levels,** pg/mg			0.86
Median (IQR)	11.47 (6.43–24.73)	9.97 (6.55–22.53)	
**Hair 20β-dihydrocortisol levels,** pg/mg			0.15
Median (IQR)	1.35 (0.69–2.90)	1.97 (1.20–3.81)	
**Ratio hair cortisol/cortisone levels**			0.21
Median (IQR)	0.22 (0.10–0.32)	0.22 (0.17–0.40)	

AI, adrenal incidentalomas; N, number of patients; IQR, interquartile range; BMI, body mass index; DST, dexamethasone suppression test; UFC, urinary free cortisol; MSC, midnight salivary cortisol; ACTH, adrenocorticotropic hormone.

Bold characters indicate statistically significant difference.

**Figure 3:**
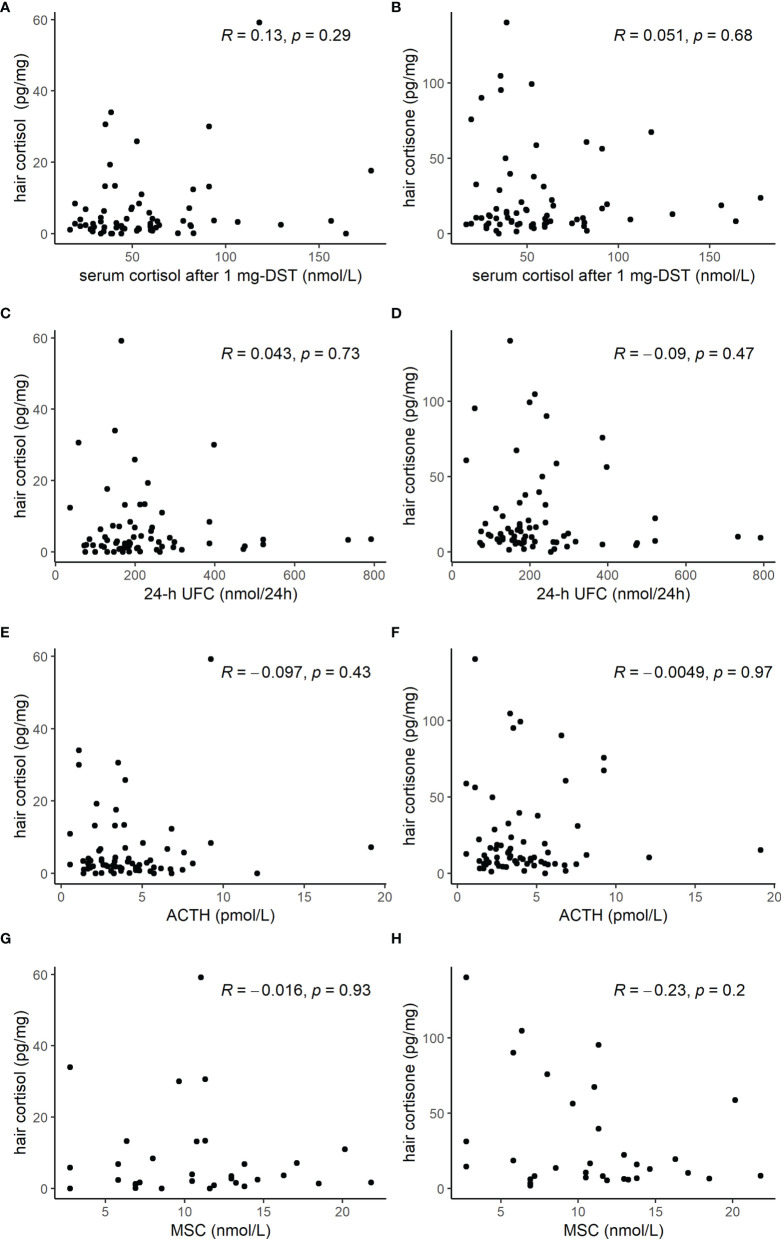
Scatterplot of hormone tests [cortisol after 1 mg dexamethasone suppression test (DST), 24-h urinary free cortisol (UFC), adrenocorticotropic hormone (ACTH), and midnight salivary cortisol (MSC)] and hair cortisol **(A, C, E, G)** or hair cortisone **(B, D, F, H)** levels, showing the lack of any significant correlation.

Hair cortisol, 20β-dihydrocortisol, and cortisone concentrations were similar in AI patients with serum cortisol after 1 mg-DST ≤ 50 nmol/l and AI patients with values > 50 nmol/l, as shown in **Table 4**. None of the three compounds in the keratin matrix showed a correlation with serum cortisol after 1 mg DST, 24-h UFC, plasma ACTH, or MSC (**Figure 3**).

**Figure 4:**
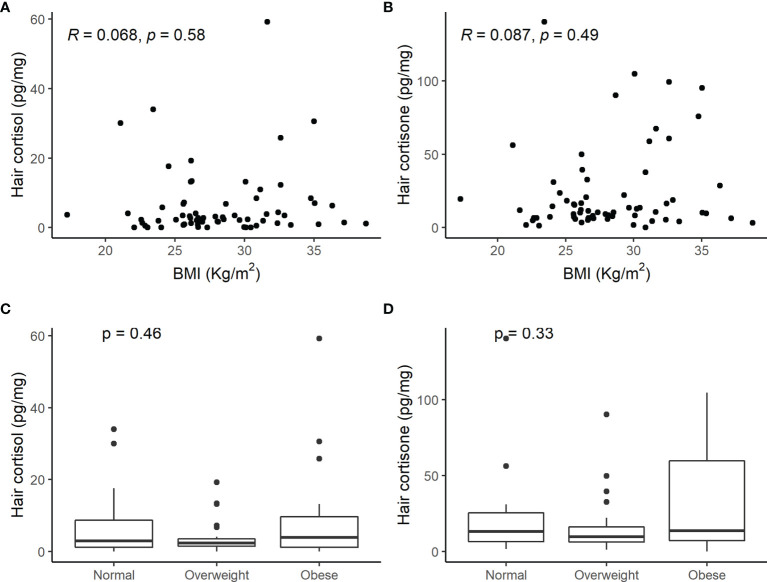
Scatterplot of hair cortisol **(A)** and hair cortisone **(B)** levels and body mass index (BMI) and comparison of hair cortisol **(C)** and cortisone **(D)** concentrations in normal weight, overweight, or obese patients with adrenal incidentaloma. No significant correlation between hair steroids and weight was observed.

Moreover, there was no correlation between cortisol and cortisone concentrations in the keratin matrix and BMI and no difference among normal weight, overweight, or obese patients (**Figure 4**).

## Discussion

This is the first study that has assessed specifically the value of hair glucocorticoid measurement as a possible diagnostic tool in patients with AI. This clinical setting is of particular interest due to the challenge in the identification of a mild cortisol excess, in a large number of patients who are indistinguishable from the general population, lacking a typical phenotype. Worthy of note is that all tests currently used for the diagnosis of cortisol excess (1 mg DST, 24-h UFC, MSC) reflect only instant or short-term secretion and cannot measure the chronic exposure to cortisol excess. For these reasons, the use of hair glucocorticoid measurement is appealing as a diagnostic test in these patients, arguing that a measure of chronic accumulation of cortisol in a tissue may improve the recognition of a minimal excess rather than using a point-like determination.

In our study, however, we did not find any difference in cortisol, cortisone, and 20β-dihydrocortisol hair concentrations between healthy controls and AI patients. Moreover, no significant difference was found in cortisol, cortisone, and 20β-dihydrocortisol levels of AI patients with or without cortisol autonomy (either ACS or PACS).

To date, several studies demonstrated a good performance of hair cortisol measurements to identify overt Cushing syndrome. Some studies conducted on a small number of patients found that hair cortisol levels were higher in patients with overt Cushing syndrome compared with healthy controls (19–21). In 2017, Wester et al. confirmed the diagnostic utility of hair cortisol in 43 patients with overt Cushing syndrome (26 with Cushing disease, 10 with adrenal Cushing, and 7 with ectopic ACTH secretion), reporting a sensitivity and a specificity of hair cortisol measurements for CS of 93% and 90%, respectively (22). In 2019, Savas et al. for the first time applied the LC-MS/MS technique for the measurements of hair cortisol and hair cortisone in a large cohort of 89 patients with endogenous Cushing syndrome, compared to 295 controls (23). Although both glucocorticoids were significantly higher in Cushing patients than in controls, hair cortisone was even most accurate in differentiating Cushing patients from healthy subjects (87% sensitivity, 90% specificity, 96% negative predictive value) than hair cortisol (81% sensitivity, 88% specificity, 94% negative predictive value) (23).

Conversely, literature on hair glucocorticoid measurement in patients with AI and cortisol autonomy is very limited. Only a study reported data on 23 patients affected by mild Cushing with 24 overt Cushing and 84 healthy subjects (24). Brossaud and colleagues reported that in patients with mild Cushing, the hair cortisol and cortisone levels were lower than in overt Cushing, but higher than in healthy controls. However, only 8 out of 23 patients with mild Cushing had a unilateral adrenocortical incidentaloma and 6 bilateral macronodular adrenal hyperplasia (BMAH), while the remainders had ACTH-dependent Cushing syndrome (24).

A possible explanation for the lack of any significant difference in hair glucocorticoids levels in our study may rely on our inadvertent inclusion of patients with a low level of cortisol secretion, since most patients had normal levels of 24-h UFC and plasma ACTH was suppressed only in few patients. Brossaud and colleagues included patients in which mild Cushing was defined as serum cortisol > 50 nmol/l after 1 mg DST and at least one of the following endocrine alterations: mildly increased UFC (≤1.5 ULN), suppressed plasma ACTH levels (< 10 pg/ml) in patients with adrenal tumours, late-night salivary cortisol or midnight serum cortisol. Another hallmark which shows that in our study the degree of cortisol autonomy in the group of AI was rather limited is that the median cortisol after 1 mg dexamethasone suppression test was of 2.5 μg/dl. However, our cohort is representative of the current series of patients with AI, with only a minimal degree of hypercortisolism (5). It may be speculated that a higher amount of cortisol excess is needed to have a significant accumulation in hair.

Interestingly, in our cohort hair cortisone levels were higher than those of cortisol, similarly to what was found in saliva (30). This finding is in line with previous studies and may be explained with the presence in the scalp of the 11β-hydroxysteroid dehydrogenase type 2 enzyme (23, 24).

The strengths of this study are the detailed characterization of the patients and the use of the UPLC-MS/MS method, which offers high specificity and accuracy together with the possibility to simultaneously analyze several target analytes. These analytical features overcome the limitations typical of immunoassay, including cross-reactivity and single-component determination (26, 31). Moreover, the high sensitivity provided by the UPLC-MS/MS method allows to conduct the analysis on small amounts of hair, which are easily collected and stored at room temperature (31).

However, we have to underline that the measurement of hair glucocorticoid levels entails pre-analytical issues. It has been demonstrated that shampoo use and hair dying can interfere with the measurement, while differences in race and age can influence cortisol incorporation in the scalp (32, 33), potentially increasing the interindividual variability and affecting the diagnostic performance of this tool.

Moreover, we should acknowledge the limits of the retrospective nature of our study and of a rather limited sample size. Although the monocentric nature of the study avoided bias due to inter-laboratory variability, it may limit the validity of our results to a specific population. Therefore, we are aware that only multicentric and prospective studies on a larger AI cohort can provide definitive results.

Despite these limits, our study provides useful evidence for the practical management of adrenal incidentalomas and assessment of cortisol autonomy.

## Conclusion

This is the first study that focused on the diagnostic use of hair cortisol and cortisone measurement in patients with incidentally discovered adrenal masses given that this is an appealing, easy-to-perform and non-invasive tool.

Our findings do not support the use of hair glucocorticoid measurement as a diagnostic test for cortisol autonomy in patients with AI.

## Data Availability Statement

The original contributions presented in the study are included in the article/supplementary material. Further inquiries can be directed to the corresponding author.

## Ethics Statement

The studies involving human participants were reviewed and approved by the Ethics Committee of San Luigi Gonzaga Hospital. The patients/participants provided their written informed consent to participate in this study.

## Author Contributions

All authors contributed to the article and approved the submitted version.

## Funding

This research was funded by Ricerca Locale Università di Torino 2020 - RILO 2020.

## Conflict of Interest

The authors declare that the research was conducted in the absence of any commercial or financial relationships that could be construed as a potential conflict of interest.

## Publisher’s Note

All claims expressed in this article are solely those of the authors and do not necessarily represent those of their affiliated organizations, or those of the publisher, the editors and the reviewers. Any product that may be evaluated in this article, or claim that may be made by its manufacturer, is not guaranteed or endorsed by the publisher.
